# Exosomal Long Non-coding RNA HOTTIP Increases Resistance of Colorectal Cancer Cells to Mitomycin via Impairing MiR-214-Mediated Degradation of KPNA3

**DOI:** 10.3389/fcell.2020.582723

**Published:** 2021-01-28

**Authors:** Xijuan Chen, Yingqiang Liu, Qinglan Zhang, Baoxing Liu, Yan Cheng, Yonglei Zhang, Yanan Sun, Junqi Liu, Hong Gen

**Affiliations:** ^1^Department of Radiation Oncology, The Affiliated Tumor Hospital of Zhengzhou University, Zhengzhou, China; ^2^Department of General Surgery, The Affiliated Tumor Hospital of Zhengzhou University, Zhengzhou, China; ^3^Department of Hematology, The Affiliated Tumor Hospital of Zhengzhou University, Zhengzhou, China; ^4^Department of Chest Surgery, The Affiliated Tumor Hospital of Zhengzhou University, Zhengzhou, China; ^5^Department of Gynecology, The First Affiliated Hospital of Zhengzhou University, Zhengzhou, China; ^6^Department of Radiation Oncology, The First Affiliated Hospital of Zhengzhou University, Zhengzhou, China

**Keywords:** colorectal cancer, exosomes, long non-coding RNA, HOXA distal transcript antisense RNA, drug resistance, microRNA-214, KPNA3

## Abstract

It has been reported that long non-coding RNA HOXA distal transcript antisense RNA (lncRNA HOTTIP) functions as a tumor promoter in colorectal cancer (CRC). Hence, we paid attention to exploring whether exosomes could carry lncRNA HOTTIP to affect the mitomycin resistance in CRC and to identify the underlying mechanisms. High expression of HOTTIP was detected in mitomycin-resistant CRC cells. Inhibition of HOTTIP reduced the mitomycin resistance. In the co-culture system of mitomycin-resistant cells or their derived exosomes with CRC cells, the HOTTIP was found to be transferred into the parental cells via extracellular vesicles (EVs) secreted from mitomycin-resistant cells and to contribute to the mitomycin resistance. Based on the bioinformatics databases, possible interaction network of HOTTIP, microRNA-214 (miR-214) and Karyopherin subunit alpha 3 (KPNA3) in CRC was predicted, which was further analyzed by dual-luciferase reporter, RNA binding protein immunoprecipitation and RNA pull-down assays. As HOTTIP down-regulated miR-214 to elevate the KPNA3 expression, HOTTIP enhanced the mitomycin resistance through impairing miR-214-dependent inhibition of KPNA3. Finally, HOTTIP was suggested as an independent factor predicting mitomycin response in patients with CRC. Those data together confirmed the promotive effects of EV-carried HOTTIP on the mitomycin resistance, while targeting HOTTIP might be a promising strategy overcoming drug resistance in CRC.

## Introduction

Colorectal cancer (CRC), most of which initiates from colorectal adenomas, is regarded as one of the frequently occurring cancers and one of the reasons of the death caused by cancer around the world (Dekker and Rex, 2018). There is still insufficient information about the global prevalence of CRC (Wong et al., 2020). Great improvements have been witnessed in the therapy of CRC such as using drugs including the anti-EGFR monoclonal antibodies cetuximab and panitumumab, as well as the multi-kinase inhibitor regorafenib and so on (De Mattia et al., 2015). Chemotherapy serves as one of selectable treatment options for patients with metastatic CRC. For example, mitomycin is a chemotherapeutic agent that shows some modest efficacy at low cost for CRC (Dimou et al., 2010). However, the chemoresistance can lead to the treatment failure (Codony-Servat et al., 2017). Clarification of the pathogenesis of CRC in terms of molecular biology and identification of biomarkers with high sensitivity/specificity have attracted our attention (Xue et al., 2020). Therefore, this study aims to identify the mechanism associated with tumorigenesis and drug resistance in CRC so as to develop novel strategies for cancer prevention.

Extracellular vesicles (EVs) are vehicles that can transfer biological signal molecules like long non-coding RNAs (lncRNAs), microRNAs (miRNAs or miRs), and messenger RNAs (mRNAs) involved in intercellular communication and have raised great interest and they are considered as biomarkers for cancer diagnosis (Pomatto et al., 2019; Sun et al., 2018a). LncRNAs are a group of RNAs that are involved in a number of biological processes via epigenetic modifications (Izquierdo and Crujeiras, 2019), and have been usually regarded as potential cancer targets (Quinn and Chang, 2016; Esposito et al., 2019). LncRNAs have been revealed to mediate a plenty of cell processes and are involved in the progression of CRC (Abedini et al., 2019). Bioinformatics analysis in microarray dataset GSE68204 suggested lncRNA HOXA distal transcript antisense RNA (HOTTIP) as a differentially expressed lncRNA between preoperative chemoradiotherapy responder and non-responder. Moreover, HOTTIP has been reported to promote CRC cell proliferation via silencing the expression of p21 (Rui et al., 2019). Another recent research has shown that knocking down HOTTIP would exert impede migrative and proliferative potentials and induce apoptotic potential of CRC cells (Liu et al., 2018a). A more recent study has characterized the prognostic value of exosomal HOTTIP in the patients with CRC that low exosomal HOTTIP level correlates with short overall survival (Oehme et al., 2019). Hence, in this study, we intended to explore whether EVs could transfer HOTTIP to mediate the drug resistance of CRC cells and the underlying mechanisms.

Long non-coding RNAs have the ability to antagonize the post-transcriptional regulation of miRNAs in expression of genes so that they play a vital role during disease development (Tay et al., 2014). miRNAs are small non-coding RNAs with a length of 18–25 nucleotides which possess the potential to regulate gene expression and are involved in cancer development (Adlakha and Seth, 2017). For example, miR-214 has been reported to act as a tumor suppressor involved in the regulation of CRC progression (He et al., 2016). Karyopherin subunit alpha 3 (KPNA3) is identified as a target gene of miR-214-3p in sinonasal inverted papilloma (Teng et al., 2018). KPNA3 is demonstrated to be capable of promoting the growth and invasiveness of hepatocellular carcinoma cells (Hu et al., 2019). In this study, we aim to determine the HOTTIP/miR-214/KPNA3 regulatory network in the drug resistance of CRC cells and to determine the role of EVs transferring RNAs involved in the resistance of CRC cells to mitomycin so as to develop novel targets overcoming drug resistance.

## Materials and Methods

### Ethics Statement

The current study was performed with the approval of the Ethics Committee of the Affiliated Tumor Hospital of Zhengzhou University and in accordance with the *Declaration of Helsinki*. Written informed consent was obtained from each participant. All animal experiments were approved by the Animal Ethics Committee of the Affiliated Tumor Hospital of Zhengzhou University and strictly followed the principle of completing the experiment with the minimum number of animals and minimizing the pain of the experimental animals.

### Study Subjects

A total of 95 patients with advanced CRC from the Affiliated Tumor Hospital of Zhengzhou University between July 2011 and May 2014 were enrolled in this study, including 46 males and 49 females (20–74 year old, median age, 43 years old). Among them, 25 cases at stage I, 46 cases at stage II, and 24 cases at stage III; 71 cases had lymph node metastasis while 24 cases without. Besides, there were three types of histological types in 95 patients: adenocarcinoma accounted for 68.42% (65/95), mucinous adenocarcinoma accounted for 25.26 (24/95), and nine patients (6.32%) were adenosquamous carcinoma. CRC tissues and adjacent normal tissues (surgical specimens or biopsies, 5 cm away from cancer tissues) were collected from patients before mitomycin treatment. Blood samples were collected one day before surgery and centrifuged at 2000 × *g* for 10 min at 4°C. The supernatant (plasma) was then sub-packaged and stored at −80°C. Patients who received at least six courses of treatment were selected as the mitomycin group, and who received no chemotherapy or who experienced mitomycin discontinuation due to adverse events (<21 days) served as controls. The response of the tumors to chemotherapy was assessed by a three-dimensional volume reduction rate or tumor response rate (radiological assessment), and evaluated according to the Response Evaluation Criteria In Solid Tumors guidelines. Patients with symptomatic deterioration, or new lesions, or tumor regeneration rate ≥25% during the verification period were included in the PD group (*n* = 40), and the remaining ones in the non-PD group (*n* = 55). PFS is defined as the duration of tumor resection to PD. All patients were followed up every 3 months (the first 2 years after treatment), 6 months (the second to fourth years after treatment), and annually until death or June 2019. Follow-up data included abdominal computed tomography and postoperative physical examination.

### Cell Culture

Colorectal cancer cell lines HCT116 and SW620 as well as human normal intestinal epithelial cell line (FHC) were obtained from American Type Culture Collection (Rockville, MD, United States). CRC cell lines including LoVo, HT29, SW480, SW1116, and Caco2 were purchased from the China Center for Type Culture Collection (Shanghai, China). The cells were cultured in Dulbecco’s modified Eagles Medium (DMEM) (GIBCO BRL, Grand Island, NY, United States) or L-15 medium containing 10% fetal bovine serum (FBS; GIBCO BRL) and 1% penicillin-streptomycin in carbon dioxide incubator at 37°C. The mitomycin- (Sigma-Aldrich, St. Louis, MO, United States) resistant CRC cell lines HCT116Mito and SW620Mito were obtained by *in vivo* xenograft and mitomycin treatment. For co-culture of EVs, 1 μg/mL EVs were added to the medium of recipient cells (5 × 10^6^). The bicinchoninic acid protein assay kit (Thermo Fisher Scientific, Waltham, MA, United States) was employed for determination of EV concentration.

### Short Hairpin RNA (shRNA) Construction

The sh-HOTTIP and scrambled control shRNA were inserted into pLVX-tdTomato-Puro lentiviral vector (Open Biosystems, Huntsville, AL, United States). The forward and reverse sequences of sh-HOTTIP were 5′-GATCCGCTGCTTTAGAGCCACATATTCAAGAGATATGTG GCTCTAAAGCAGCTTTTTTCTCGAGG-3′ and 5′-AATTC CTCGAGAAAAAAGCTGCTTTAGAGCCACATATCTCTTGA ATATGTGGCTCTAAAGCAGCG-3′, respectively. The forward and reverse sequences of scrambled control shRNA were 5′-CCGGTTTCTCCGAACGTGTCACGTCTCGAGACGTGACAC GTTCGGAGAATTTTTG-3′ and 5′-AATTCAAAAAGTTCTC CGAACGTGTCACGTCTCGAGACGTGACACGTTCGGAGAA-3′, respectively.

Stable HOTTIP knockdown cells were generated by infection with lentivirus harboring shRNA. Then, 72 h after the 239 T cells were transduced with lentiviral, the lentivirus supernatants were harvested. The lentiviruses were concentrated overnight at 4°C using a Lenti-X^TM^ concentrator (Clontech, Mountain View, CA, United States) and then stored at −80°C. The virus titer of the concentrated particles was 1.1 × 10^8^ TU/mL. HCT116Mito and SW620Mito cells (5 × 10^5^ cells/well) were seeded in 6-well culture dishes. The cells were cultured in the DMEM containing 10% FBS for 24 h before infection. Then, 72 h after infection, the cells were treated with Puromycin (10 μg/mL) to screen the stably infected cell line for 2–3 weeks in total with the medium renewed every 2–3 days. siHOTTIP, siKPNA3, siCtrl, HOTTIP overexpression plasmid (pcDNA3.1-HOTTIP), KPNA3 overexpression plasmid (pcDNA3.1-KPNA3) and empty vector were purchased from Sigma-Aldrich. miR-214 mimic, sponge and control were produced by GeneChem (Shanghai, China). HCT116 and SW620 cells (1 × 10^5^/well) were seeded in 6-well plates and then transfected with the aforementioned siRNA, plasmid or vector at a final concentration of 100 nM using Lipofectamine 2000 reagent (Thermo Fisher Scientific).

### Reverse Transcription-Quantitative Polymerase Chain Reaction (RT-qPCR)

RNA (30 μl) was exacted from cells (1 × 10^5^ cells) or tissues (30 mg) using TRIzol kit (Invitrogen, Grand Island, NA, United States) according to the manufacturer’s instructions. The value of OD260/OD280 of RNA was between 1.8 and 2.0, and two obvious peaks of 18s and 28s can be seen through capillary electrophoresis (Supplementary Figure 1A). A RT kit (RR047A, Takara, Japan) was used for reverse transcription of mRNA and lncRNA. miRNA was subjected to RT using miRNA First Strand cDNA Synthesis (Tailing Reaction) kit (B532451-0020, Sangon Biotechnology Co., Ltd., Shanghai, China) to obtain cDNA. Real-time PCR was performed using the iTaq^TM^ universal SYBR Green I Kit (Bio-Rad, Hercules, CA, United States). RNA in culture medium or plasma was extracted by mirVana PARIS Kit (Ambion, Carlsbad, CA, United States). With Glyceraldehyde-3-phosphate dehydrogenase (GAPDH) and U6 as internal reference, the RNA levels in condition medium (CM), plasma and EVs were normalized against a synthesized exogenous reference λ polyA + RNA (Takara). All primers shown in Table 1 were synthesized by Guangzhou RiboBio Biotechnology Co., Ltd. (Guangzhou, China).

**TABLE 1:** Primer sequences for reverse transcription quantitative polymerase chain reaction.

Gene	Primer sequence (5′-3′)
HOTTIP	F: 5′-AGCTCTCAGGGAAACGAAGC-3′
	R: 5′-TTTCCGGCAAACTCCCTCTC-3′
miR-214	F: 5′-AGCCGACAGCAGGCACAGACA-3′
	R: 5′-GCTTCGGCAGCACATATACTAAAAT-3′
KPNA3	F: 5′-AGCTCCTGGGCGTCATCATA-3′
	R: 5′-AGGTCTGCGATCTCGGCTTT-3′
GAPDH	F: 5′-TCATCTCTGCCCCCTCTGCTG-3′
	R: 5′-GCCTGCTCACCACCTTCTTG-3′
U6	F: 5′-CGCTTCGGCAGCACATATACTA-3′
	R: 5′-CGCTTCACGAATTTGCGTGTCA-3′

*HOTTIP, HOXA distal transcript antisense RNA; miR-214, microRNA-214; KPNA3, karyopherin subunit alpha 3; GAPDH, glyceraldehyde-3-phosphate dehydrogenase; F, forward; R, reverse.*

### Western Blot Analysis

Cellular proteins were extracted using radioimmunoprecipitation assay protein extraction reagent (Beyotime Biotechnology Co., Shanghai, China) containing a mixture of protease inhibitors and phenylmethylsulfonyl fluoride (Roche, Basel, Switzerland). And 30 μg protein was separated with 10% sodium dodecyl sulfate-polyacrylamide gel electrophoresis. Then the protein was transferred to a polyvinylidene fluoride membrane (EMD Millipore, Billerica, MA, United States), and blocked with 5% skim milk for 2 h. The membranes were incubated with specific antibodies at 4°C overnight, including rabbit anti-CD63 (1: 1000, ab134045, Abcam, Cambridge, United Kingdom), rabbit anti-CD9 (1: 1000, ab263019, Abcam), rabbit anti-GM130 (1: 1000, ab215966, Abcam), and rabbit anti-KPNA3 (1: 1000, ab137446, Abcam). GAPDH (1: 5000, ab181602, Abcam) was used as a control. Protein bands were visualized using the Millipore enhanced chemiluminescence Western Blotting Detection System (Millipore). The experiment was conducted in triplicates for three times independently.

### Cell Counting Kit-8 (CCK-8) Assay

In total, 2 × 10^3^ HCT116Mito or SW620Mito cells (or parental cells) were seeded in 96-well plates for 12 h. Cells were treated with different concentrations of mitomycin for 72 h. Cell viability was examined using CCK-8 assay (Dojindo Molecular Technologies, Kyushu, Japan). Briefly, the cells were incubated with 10 μL CCK-8 solution at 37°C for 2 h. The optical density was detected at 450 nm (Wellscan MK3; Labsystems Dragon, Finland). The experiment was conducted in triplicates for three times independently.

### Colony Formation Assay

The cells (500 cells/well) were seeded in 6-well plates. The next day, the cells were treated with mitomycin (5 μg/mL) for 24 h, and then the medium was renewed with mitomycin-free medium. After 14 days, the cells were fixed with 4% paraformaldehyde, stained with 0.1% crystal violet and the formed colonies were counted. The experiment was conducted in triplicates for three times independently.

### Flow Cytometry

After transfection, the cells were washed and resuspended in 1 × binding buffer (BD Biosciences, San Jose, CA, United States). Cells were stained with Annexin V-fluorescein isothiocyanate and propidium iodide (5 μg/mL) for 15 min in the dark, and cell apoptosis was detected on flow cytometer (Becton-Dickinson, Franklin Lakes, NJ, United States).

### Transwell Assay

The cells (2 × 10^4^) were cultured in a Matrigel-coated apical chamber (8 μm, BD Biosciences). The medium without FBS was added to the apical chamber, and Roswell Park Memorial Institute 1640 medium containing 20% FBS was added to the basal chamber. The cells were incubated for 24 h at 37°C with 5% CO_2_. The apical chamber was cleaned with a cotton swab, and the basolateral chamber was washed with phosphate buffered saline (PBS). The cells were fixed with 4% paraformaldehyde, stained with 0.1% crystal violet, washed three times with water, observed and counted with inverted microscope (Carl Zeiss, Jena, Germany). The cell migration was tested similarly as Transwell assay except that the chamber was not coated with Matrigel.

### Immunofluorescence, Immunohistochemistry and Fluorescence *in situ* Hybridization (FISH)

After being cultured for 24 h, the cells were fixed with 4% paraformaldehyde for 15 min at ambient temperature. After PBS washes, the cells were permeabilized in PBS containing 0.1% Triton X-100, blocked with 10% normal goat or sputum serum in 1% bovine serum albumin/PBS for 1 h and then incubated with primary antibody rabbit anti-γ-H2AX (ab2893, 1: 500, Abcam) at 4°C overnight. The cells were then incubated with Alexa Fluor^®^ 647-conjugated secondary antibody goat anti-rabbit immunoglobulin G (IgG) (ab150075, 1: 500, Abcam) for 1 h, and washed with PBS for 3 times again. Finally, the cells were stained with Vectashield containing 4′,6-Diamidino-2-Phenylindole (DAPI) and observed under confocal laser scanning microscope.

Paraffin-embedded sections were deparaffinized and rehydrated, followed by antigen retrieval. After incubation with the primary antibody rabbit anti-KPNA3 (1: 100, ab137446, Abcam) and secondary antibody horseradish peroxidase-conjugated IgG (1: 1000, ab6721, Abcam), the sections were finally incubated with diaminobenzidine (DAKO, Glostrup, Denmark) and counter-stained with hematoxylin (Sigma-Aldrich).

Fluorescence *in situ* hybridization was performed according to the instructions of microRNA FISH Probe (FI-0013H, Exon Biotechnology Co., Ltd., Guangzhou, China) and Ribo^TM^ lncRNA FISH Probe Mix (Red) (C10920, Guangzhou RiboBio Biotechnology, Co., Ltd.). The cells were fixed with 1 mL 4% paraformaldehyde for 10 min, and permeabilized with 1 mL pre-cooled PBS containing 0.5% Triton X-100 for 5 min at 4°C. Next, the cells were blocked with 200 μL pre-hybridization solution per well at 37°C for 30 min and then hybridized with anti-HOTTIP nucleotide probe hybridization solution (Wuhan GeneCreate Biological Engineering Co., Ltd., Wuhan, Hubei, China) overnight at 37°C. After washing, cells were stained with DAPI (1: 800) for 10 min, and sealed with nail polish. Five different fields of view were selected for observation and photographing under fluorescence microscope (OLYMPUS, Tokyo, Japan).

### RNA Binding Protein Immunoprecipitation (RIP) Assay

RNA binding protein immunoprecipitation experiments were conducted according to the manual of RIP^TM^ RNA-Binding Protein Immunoprecipitation Kit (Millipore). Cell lysates were incubated with specific antibody against Ago2 (1: 50, Merck Millipore) or normal mouse IgG (Millipore) in the negative control (NC) and RIP buffer containing magnetic beads. Immunoprecipitated RNA was determined by RT-qPCR.

### RNA Pull-Down

Biotin-labeled RNA was transcribed using T7 or SP6 RNA polymerase (Promega, Madison, WI, United States) and labeled with Biotin RNA Labeling Mix (Roche). Biotinylated RNAs were treated with RNase-free DNase I (Roche) and purified with RNeasy Mini Kit (QIAGEN, Düsseldorf, Germany). The cell lysate was incubated with biotinylated RNA for 1 h and then with streptavidin agarose beads (Invitrogen) for 1 h. Precipitates were washed for five times. RNA was purified, and then extracted by TRIzol method, followed by RT-qPCR determination.

### Isolation and Identification of EVs

When the cell confluence reached 70–80%, the cells were cultured with DMEM supplemented with 10% FBS deprived of EVs. After 48 h, the cell supernatant was centrifuged at 300 × *g* for 10 min and centrifuged at 2000 × *g* for 10 min to remove dead cells and cell debris. The supernatant was filtered through a Steritop^TM^ 0.22 μm sterile filter (Millipore), and centrifuged at 100000 × *g* for 2 h. The collected pellet was resuspended in 15 mL PBS and centrifuged at 4000 × *g* in a centrifugal filter until the final volume was reduced to 200 μL. The above steps were all carried out at 4°C. Isolated EVs were used immediately or stored at −80°C for subsequent analysis. To observe the EVs, the exosomal suspension was diluted with PBS, and fixed with 2% paraformaldehyde for 30 min, followed by resuspension in PBS and fixation with 2% paraformaldehyde for another 30 min. A total of 8 μL sample was dropped onto an Em grid pretreated with ultraviolet light. After drying for 30 min, the EVs were stained twice with 1% uranyl acid (6 min each time). Finally, observation was performed at 120 kV using a H-7650 transmission electron microscope (TEM; Hitachi, Tokyo, Japan).

Quantitative analysis of EV diameter was performed by NanoSight NS300 instrument (Malvern Instruments Ltd., United Kingdom) equipped with nanoparticle tracking analysis (NTA) 3.0 analysis software (Malvern Instruments). Each sample was tracked with a camera for 30 s, analyzed with NanoSight version 2.3 (NanoSight Ltd., Amesbury, United Kingdom) software. With gain of all samples 6.0 and the threshold 11, the particle trajectory was recorded and the sample dilution concentration was detected, along with particle size distribution graph. Then the EV concentration of the stock solution was calculated according to the dilution ratio.

The expression of EV surface markers CD9, CD63, and *cis-*Golgi matrix protein GM130 was measured by Western blot analysis.

### Co-culture of CRC Cells and EVs

A total of 400 nM fluorescence labeled HOTTIP were mixed in 400 μL electroporation buffer and electroporated into 2 μg EVs in 4 mm tubes at 350 V and 150 μF using a Gene Pulser Xcell^TM^ electroporation system (Bio-Rad). The mixture was incubated at 37°C for 30 min and then treated with RNase to remove unbound RNAs. Fluorescence was measured using a spectrophotometer (F-4600; Hitachi) to quantify the labeled RNA in EVs.

The EVs were incubated with 1 μM Dil (Beyotime) for 20 min, and then centrifuged with spin column (MW3000; Invitrogen). The CRC cells were co-cultured with Dil-labeled EVs and observed under a fluorescence microscope after 24 h of co-culture.

### Dual Luciferase Reporter Assay

Karyopherin subunit alpha 3 3′UTR gene fragment containing putative binding site of miR-214 was introduced into psiCHECK2 vector (Promega). The MUT form, in which the binding site was mutated, was designed based on the seed sequence KPNA3 WT. The MUT gene fragment was also inserted into the psiCHECK2 reporter plasmid. The WT and MUT luciferase reporter plasmids were respectively co-transfected with miR-214 mimic into the HEK-293T cells (CRL-1415, Shanghai Xinyu Biological Technology Co., Ltd., Shanghai, China). After 48 h, the cells were collected and lysed, and the luciferase activity was detected on Glomax 20/20 luminometer (Promega) using a luciferase activity assay kit (RG005, Beyotime). The same method was used to detect the relationship between HOTTIP and miR-214.

### *In vivo* Chemosensitivity Assessment

Forty BALB/c nude mice (aged 6–8 weeks) were subcutaneously injected with HCT116Mito cells (5 × 10^6^ cells/mouse) transfected with shRNA or sh-HOTTIP, or injected with HCT116 cells (5 × 10^6^ cells/mouse) transfected with oe-HOTTIP or Ctrl plasmid, respectively, in the dorsal side. The remaining 20 mice were injected with PBS, HCT116-derived EVs (HCT116 exos), HCT116Mito derived EVs (HCT116Mito exos) or HCT116-derived EVs electroporated with HOTTIP (HCT116 exos HOTTIP electrop), every 3 days with 5 μg per injection. Those nude mice were intravenously treated with mitomycin (8.8 mg/kg) or NaCl once a week when xenografts reached an average size of 0.1–0.15 cm^2^ on the 5th day after cell injection, five times in total. Tumors were measured every 3–4 days and tumor volume was calculated as follows: volume = (L × W^2^)/2, where L and W are the longest and shortest diameters, respectively. The mice were euthanized when the average L was close to 1 cm.

### Statistical Analysis

SPSS version 21.0 (IBM SPSS Statistics, Chicago, IL, United States) was applied for statistical analysis. The measurement data was expressed by mean ± standard deviation, and the paired data obeying the normal distribution and the homogeneity of variance were compared between two groups with paired *t*-test, the unpaired data obeying the normal distribution and homogeneity of variance was performed using unpaired *t* test. Data among multiple groups were compared with one-way analysis of variance (ANOVA) followed by Tukey’s *post hoc* test. Data comparison between groups at different time points was performed by repeated measures ANOVA, with Bonferroni *post hoc* test. Pearson correlation analysis was used to analyze the relationship between the two indicators. A value of *p* < 0.05 was considered to be statistically significant.

## Results

### High Expression of HOTTIP Is Observed in Mitomycin-Resistant CRC Cells

**FIGURE 1:**
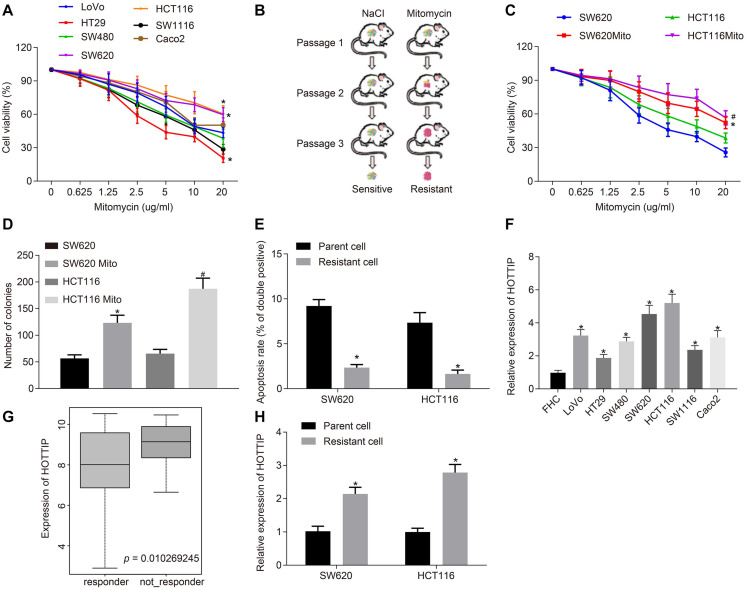
HOTTIP is highly expressed in mitomycin-resistant CRC cells. **(A)** Viability of 7 CRC cell lines (LoVo, HT29, SW480, SW620, HCT116, SW1116, and Caco2) assayed using CCK-8 after 72 h of mitomycin treatment at the indicated concentrations. **(B)** Schematic diagram of the screen process of mitomycin-resistant CRC cells. **(C)** Viability of parental cells and mitomycin-resistant cells assayed using CCK-8 after 12 h of mitomycin treatment at the indicated concentrations. **(D)** Colony formation of parental and mitomycin-resistant cells. **(E)** Apoptosis rates of parental and mitomycin-resistant cells treated with mitomycin (5 μg/mL) for 24 h determined by flow cytometry. **(F)** RT-qPCR determination of HOTTIP expression in 7 CRC cell lines (LoVo, HT29, SW480, SW620, HCT116, SW1116, and Caco2) and normal FHC cells. **(G)** Expression of the HOTTIP in the non-responder and responder based on CRC gene dataset GSE68204. **(H)** RT-qPCR determination of HOTTIP expression in parental and mitomycin-resistant CRC cells. *p < 0.05 vs. the LoVo, SW620 cells, parental cells or FHC cells; #*p* < 0.05 vs. the HCT116 cells. Data (mean ± standard deviation) between two groups were analyzed by unpaired *t* test and those among multiple groups were compared using one-way ANOVA with Tukey’s *post hoc* test. Data between groups at different time points were compared using repeated measures ANOVA, followed by Bonferroni *post hoc* test. The cell experiments were independently conducted in triplicates. *n* = 5 in animal experiments.

To select mitomycin-resistant cell lines, the seven CRC cell lines were treated with mitomycin, and the two cell lines HCT116 and SW620 with the highest half maximal inhibitory concentration (IC_50_) were chosen for the following experiments (Figure 1A). Subsequently, nude mice were inoculated with HCT116 and SW620 cells and treated with mitomycin to evaluate the drug resistance of those two cell lines (Figure 1B). The xenografted HCT116 and SW620 cells at passage 3 showed resistance to mitomycin treatment. Hence, HCT116 and SW620 cells resistant to mitomycin were isolated from these xenografts and designated as HCT116Mito and SW620Mito, respectively. For the purpose of assessing the resistance to mitomycin *in vitro*, HCT116Mito and SW620Mito cells were treated with mitomycin (5 μg/mL) for 24 h. Compared with the parental cells, HCT116Mito and SW620Mito cells responded poorly to mitomycin, as evidenced by increased IC_50_ after mitomycin treatment, enhanced anchorage-independent growth, and reduced mitomycin-induced cell apoptosis (Figures 1C–E). Moreover, mitomycin-resistant cells exhibited higher migration and invasion abilities than parental cells (Supplementary Figure 1B). Using γ-H2AX expression as an indicator of DNA double-strand break, immunofluorescence analysis showed that mitomycin induced greater DNA damage to the parental cells as compared to mitomycin-resistant cells (Supplementary Figure 1C). The above results indicated that mitomycin-resistant CRC cell line was successfully established.

Next, RT-qPCR showed that the expression of HOTTIP in seven CRC cell lines was higher than in normal intestinal epithelial cells, and we found that the expression of HOTTIP in HCT116 and SW620 cells was lower than that in other CRC cell lines (Figure 1F). The CRC-related gene expression dataset GSE68204 was downloaded from the Gene Expression Omnibus database^1^. The lncRNA expression profiling of this dataset in tumor tissues of CRC responders and non-responders revealed that HOTTIP was expressed at a higher level in the preoperative chemoradiotherapy non-responder group as compared to the responder group (Figure 1G). Consistently, the expression of HOTTIP was markedly higher in mitomycin-resistant cells than in parental cells (Figure 1H). The above results revealed that HOTTIP was highly expressed in mitomycin-resistant CRC cells and hence selected for following experiment.

### Silencing of HOTTIP Reduces Resistance of CRC Cells to Mitomycin

**FIGURE 2:**
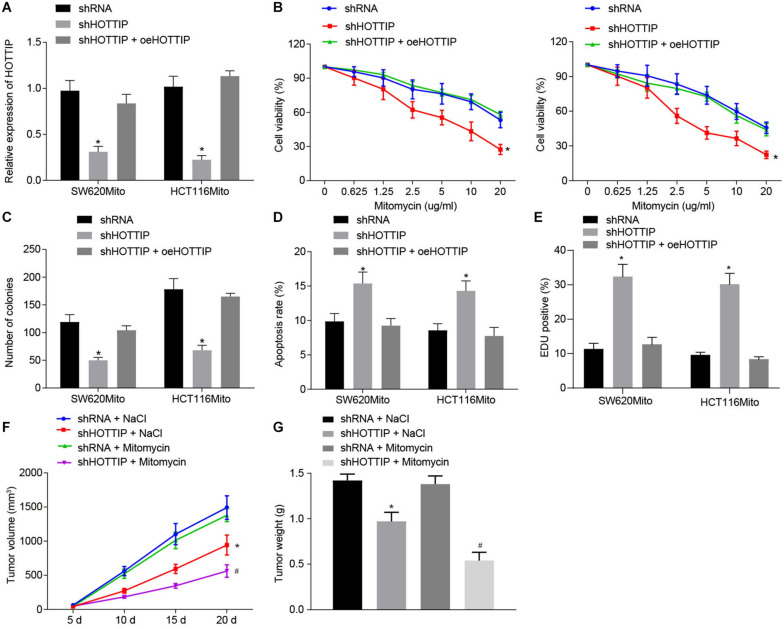
Inhibition of HOTTIP expression reduces the mitomycin resistance in CRC cells. **(A)** The expression of HOTTIP in HCT116Mito and SW620Mito cells transduced with lentiviral-mediated sh-HOTTIP, shRNA or shHOTTIP + oeHOTTIP determined by RT-qPCR. **(B)** Viability of HCT116Mito and SW620Mito cells transduced with lentiviral-mediated sh-HOTTIP, shRNA or shHOTTIP + oeHOTTIP assessed by CCK-8 assay. **(C)** Colony formation rate in HCT116Mito and SW620Mito cells transduced with lentiviral-mediated sh-HOTTI, shRNA or shHOTTIP + oeHOTTIP. **(D)** Flow cytometric analysis of apoptosis rate of HCT116Mito and SW620Mito cells transduced with lentiviral-mediated sh-HOTTIP, shRNA or shHOTTIP + oeHOTTIP. **(E)** Immunofluorescence analysis of γ-H2AX expression in cells. **(F)** Tumor volume in nude mice injected with HCT116Mito cells treated by mitomycin (8.8 mg/kg) or NaCl one time weekly, five times in total, when the volume of xenograft reached 0.1–0.15 cm^2^ on the day 5. **(G)** Tumor weight in nude mice injected with HCT116Mito cells. In **(F,G)** HCT116Mito cells (5 × 10^6^) transduced with lentiviral expressing sh-HOTTIP subcutaneously injected into the mice, and the mice were intravenously administered with mitomycin (8.8 mg/kg) once a week. *p < 0.05 vs. the cells transduced with lentiviral expressing shRNA or nude mice injected with shRNA-infected cells and NaCl; #*p* < 0.05 vs. nude mice injected with cells transduced with lentiviral expressing sh-HOTTIP and mitomycin. Data (mean ± standard deviation) between two groups were analyzed by unpaired *t* test, and among multiple groups were compared by one-way ANOVA with Tukey’s *post hoc* test. Data between groups at different time points were compared using repeated measures ANOVA with Bonferroni *post hoc* test. The cell experiments were independently conducted in triplicates. *n* = 5 in animal experiments.

To further elucidate the functional role of HOTTIP in mitomycin resistance, we stably knocked down HOTTIP expression in mitomycin-resistant cells HCT116Mito and SW620Mito via lentivirus-mediated shRNA and after confirmation of inhibitory activity, overexpression of HOTTIP was treated with the cells (Figure 2A). By evaluating the response of mitomycin-resistant cells, inhibition of HOTTIP was witnessed to cause reduced IC_50_, attenuated anchorage-independent growth, and increased mitomycin-induced cell apoptosis; however, when HOTTIP expression was restored, growing ability of cells was recovered (Figures 2B–D). Meanwhile, γ-H2AX immunofluorescence analysis showed that inhibition of HOTTIP enhanced DNA damage of mitomycin-treated cells but the injury was alleviated in the presence of oe-HOTTIP (Figure 2E).

On the other hand, we overexpressed HOTTIP in the parental cells (Supplementary Figure 2A), followed by assessment on cellular functions. It was found that overexpression of HOTTIP in the parental cells increased IC_50_, enhanced anchorage-independent growth, and reduced mitomycin-induced cell apoptosis (Supplementary Figures 2B–D). γ-H2AX immunofluorescence analysis revealed that overexpression of HOTTIP in parental cells attenuated DNA damage caused by mitomycin (Supplementary Figure 2E).

Finally, to verify whether HOTTIP could affect the resistance to mitomycin *in vivo*, HOTT116Mito cells with HOTTIP stably knocked down or HCT116 cells stably overexpressing HOTTIP were subcutaneously implanted into nude mice, which were then treated with mitomycin. It was displayed that knockdown of HOTTIP noticeably reduced mitomycin resistance in HCT116Mito xenografts *in vivo*, while overexpression of HOTTIP appreciably enhanced resistance to mitomycin (Figures 2F,G and Supplementary Figures 2F,G). Collectively, these results indicated that HOTTIP knockdown reduced the resistance of CRC cells to mitomycin.

### HOTTIP Acts as a Regulator of miR-214 to Elevate KPNA3 Expression

**FIGURE 3:**
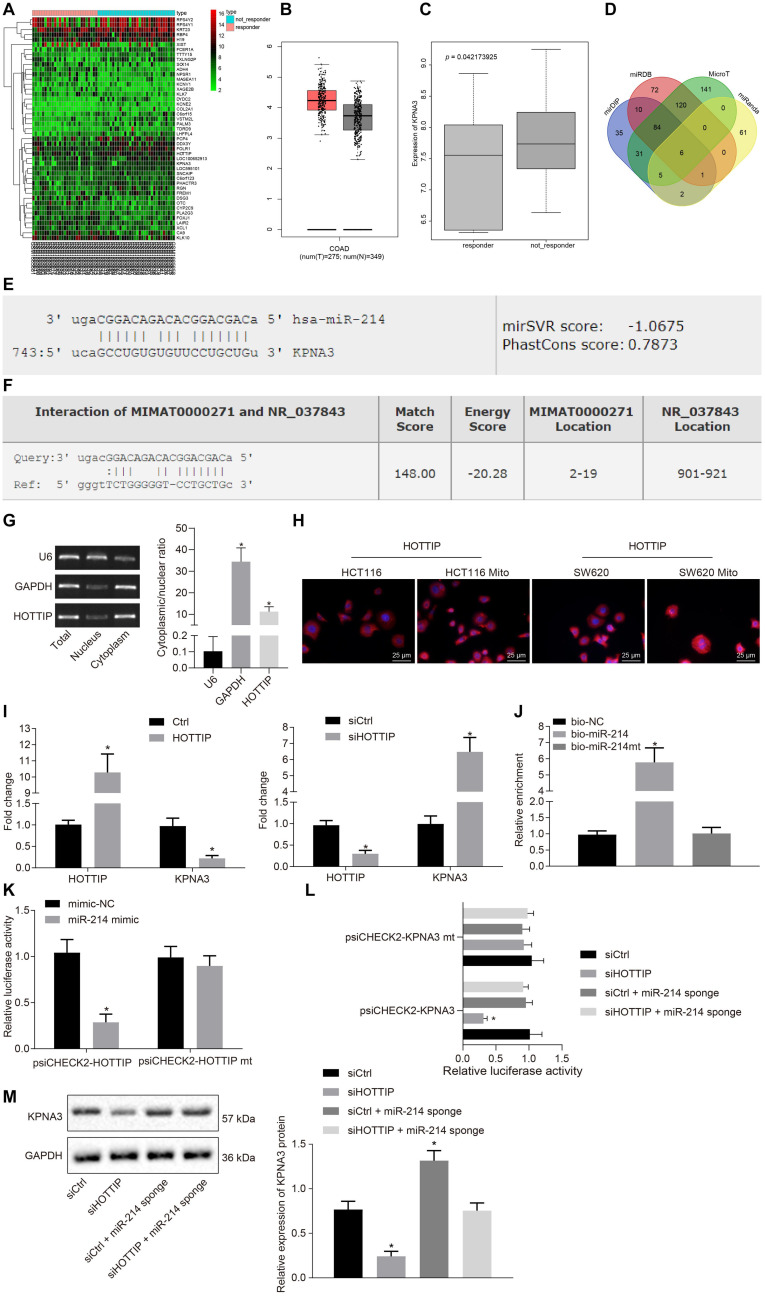
HOTTIP acts as a ceRNA of miR-214 to elevate the KPNA3 expression. **(A)** Heat map of DEGs from GSE86204 dataset. Abscissa indicates sample number, and ordinate indicates DEGs in CRC drug resistance group. **(B)** Expression of KPNA3 in CRC samples and paired normal tissues from TCGA database. **(C)** Differential expression of KPNA3 in the non-responders and responders of dataset GSE68204. **(D)** The miRNAs that could regulate KPNA3 predicted in the miDIP, miRDB, MicroT and miRanda databases, and the numbers represented the number of predicted miRNAs. **(E)** The miR-214 binding site on KPNA3. **(F)** miR-214 binding site on HOTTIP. **(G)** Subcellular localization of HOTTIP in HCT116Mito cells by RT-qPCR, with U6 and GAPDH as nuclear and cytoplasmic markers. **(H)** Immunofluorescence analysis showing HOTTIP expression and localization in HCT116 cells, HCT116Mito cells, SW620 cells, and SW620Mito cells (scale bar = 25 μm). **(I)** RIP analysis of HOTTIP and KPNA3 enriched by Ago2 when HCT116 cells were transfected with pcDNA3.1-ctrl or pcDNA3.1-HOTTIP, and HCT116Mito cells were transfected with control or si-HOTTIP. **(J)** RNA pull-down assay for the relationship between HOTTIP and miR-214 in HCT116 cells. **(K)** The binding of HOTTIP to miR-214 analyzed by dual luciferase reporter assay. **(L)** The binding of HOTTIP or KPNA3 to miR-214 analyzed by dual-luciferase reporter assay. **(M)** The KPNA3 protein expression in response to silencing of HOTTIP and/or miR-214 sponge normalized to GAPDH measured by Western blot analysis. *p < 0.05 vs. the cells transfected with Ctrl, cells transfected with siCtrl, cells transfected with bio-NC or mimic-NC. Data (mean ± standard deviation) between two groups were analyzed by unpaired *t* test, and those multiple groups were compared using one-way ANOVA with Tukey’s *post hoc* test. The cell experiments were independently conducted in triplicates.

The gene expression profiling of the dataset GSE68204 in CRC responders and non-responders was analyzed to screen differentially expressed genes (DEGs), from which 45 DEGs were obtained accordingly, of which 28 genes were up-regulated, while 17 genes were down-regulated in CRC. The 45 DEGs were clustered and a heat map of which was plotted (Figure 3A). Through the Gene Expression Profiling Interactive Analysis online website^2^, differential expression analysis of DEGs in all cancers in The Cancer Genome Atlas (TCGA) database was conducted, which revealed that KPNA3 was highly expressed in CRC (Figure 3B). Based on differential analysis of the dataset GSE68204, higher expression of KPNA3 was observed in the non-responder group than in the responder group (Figure 3C).

Next, through predicting the miRNAs that could regulate KPNA3 based on the mirDIP database^3^, the miRDB database^4^, the MicroT database^5^ and the miRanda database^6^, six intersection miRNAs with the binding site on KPNA3 were found (Figure 3D). As miR-214 has been suggested as a potential marker to predict survival for CRC patients (Chen et al., 2014) and miR-214 expression has been indicated to be a down-regulated miRNA in CRC (Wu et al., 2018), we selected miR-214 for the subsequent study. The binding sites of miR-214 on KPNA3 are shown in Figure 3E. Through the RAID v2.0 database^7^, it was predicted that HOTTIP could bind to miR-214 (Figure 3F). These results suggested that HOTTIP was highly likely to exert regulatory functions on miR-214 and KPNA3, thus affecting the drug resistance in CRC.

Since HOTTIP was primarily concentrated in the cytoplasm (Figures 3G,H), it may act as a competing endogenous RNA (ceRNA) to sequester a miRNA, thereby releasing the corresponding miRNA-targeted transcripts. To confirm our hypothesis, we performed RNA binding protein immunoprecipitation (RIP) with Argonaute2 (Ago2), a core component of the RNA-induced silencing complex. Overexpression of HOTTIP in HCT116 cells resulted in increased enrichment of HOTTIP by Ago2, but a significant reduction in enrichment of KPNA3 transcripts. In addition, knockdown of HOTTIP in HCT116Mito cells caused a significant increase in Ago2-recruited KPNA3 transcripts (Figure 3I). These results indicated that HOTTIP could compete with KPNA3 transcripts for Ago2-based miRNA-induced silencing complexes. RNA pull-down assay showed that HOTTIP was enriched in the cells transfected with biotinylated-miR-214 (bio-miR-214) in comparison to the cells transfected with bio-negative control (NC), indicating a specific interaction between HOTTIP and miR-214 (Figure 3J). Dual-luciferase reporter assay for identification on binding relationship between HOTTIP and miR-214 displayed that transfection of miR-214 mimic inhibited luciferase activity of psiCHECK2-HOTTIP, whereas the luciferase activity of psiCHECK2-HOTTIPmt could not be affected by miR-214 mimic (Figure 3K), suggesting that HOTTIP could bind to miR-214. Subsequently, we analyzed whether KPNA3 could be mediated by HOTTIP via miR-214. When HOTTIP was knocked down, the luciferase activity of KPNA3-3′untranslated region-wild type (3′UTR-WT) was reduced, which was rescued by miR-214 sponge, while the luciferase activity of KPNA3-3′UTR-mutant (MUT) remained unchanged (Figure 3L). This revealed that HOTTIP positively regulated KPNA3 via binding to miR-214. Their interactions were also confirmed at the protein levels (Figure 3M). In summary, these data indicated that HOTTIP acted as a molecular sponge of miR-214, thereby increasing KPNA3 expression.

### HOTTIP Mediates Resistance of CRC Cells to Mitomycin via MiR-214/KPNA3 Axis

**FIGURE 4:**
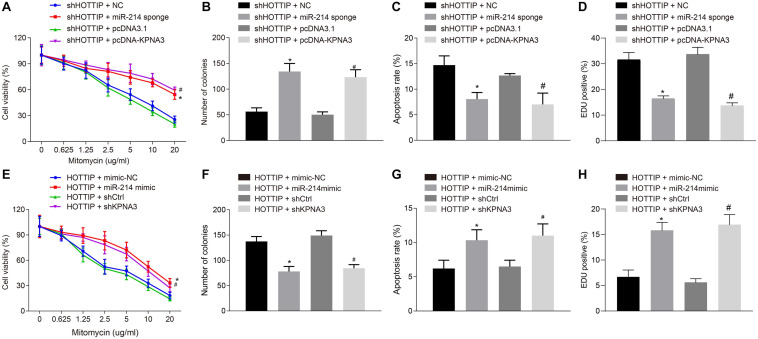
HOTTIP mediates resistance of CRC cells to mitomycin via miR-214/KPNA3 axis. **(A)** CCK-8 assay showing HCT116Mito cell activity. **(B)** HCT116Mito cell colony formation rate. **(C)** Flow cytometric analysis showing apoptosis rate of HCT116Mito cells. **(D)** Immunofluorescence analysis for γ-H2AX expression in HCT116Mito cells. **(E)** HCT116 cell viability assessed by CCK-8 assay. **(F)** Colony formation rate in HCT116 cells. **(G)** Apoptosis rate of HCT116 cells measured by flow cytometric analysis. **(H)** Immunofluorescence analysis of γ-H2AX expression in HCT116 cells detected by immunofluorescence analysis. *p < 0.05 vs. the cells co-transfected with sh-HOTTIP and NC or cells co-transfected with HOTTIP and mimic-NC; #*p* < 0.05 vs. the cells co-transfected with sh-HOTTIP and pcDNA3.1 or cells co-transfected with HOTTIP and siCtrl. Data (mean ± standard deviation) were compared by unpaired *t* test between two groups, and one-way ANOVA with Tukey’s *post hoc* test among multiple groups. Data between groups at different time points was compared using repeated measures ANOVA with Bonferroni *post hoc* test. The cell experiments were independently conducted in triplicates. *n* = 5 in animal experiments.

Next, we attempted to determine whether HOTTIP regulated mitomycin resistance of CRC cells through the miR-214/KPNA3 axis. It was found out that compared with the HCT116Mito cells co-transfected with small interfering RNA (siRNA) against HOTTIP (siHOTTIP) and siNC, the HCT116Mito cells co-transfected with siHOTTIP and miR-214 sponge exhibited increased IC_50_ (Figure 4A), enhanced anchorage-independent growth ability (Figure 4B), decreased mitomycin-induced apoptosis (Figure 4C), and weakened DNA damage (Figure 4D). Resistant phenotypic of HCT116Mito cells co-transfected with siHOTTIP and pcDNA-KPNA3 was rescued compared to the HCT116Mito cells co-transfected with both siHOTTIP and pcDNA3.1 (Figures 4A–D). Besides, HCT116 cells co-transfected with HOTTIP and miR-214 mimic had lower IC_50_, lower anchorage-independent growth ability, increased mitomycin-induced apoptosis and DNA damage compared with the cells co-transfected with HOTTIP and mimic-NC. Compared with the cells co-transfected with HOTTIP and control siRNA (siCtrl), the cells co-transfected with HOTTIP and siKPNA3 showed reduced IC_50_, decreased anchorage-independent growth, enhanced mitomycin-induced apoptosis and DNA damage (Figures 4E–H). KPNA3 knockdown in HCT116mito cells significantly decreased IC_50_, weakened anchorage-independent growth ability and stimulated mitomycin-induced apoptosis, whereas KPNA3 overexpression exerted opposite effect (Supplementary Figure 3). The above results demonstrated that HOTTIP enhanced the resistance of CRC cells to mitomycin via impairing miR-214-dependent inhibition of KPNA3.

### HOTTIP Can Be Transferred From Resistant Cells to Parental Cells via EVs

**FIGURE 5:**
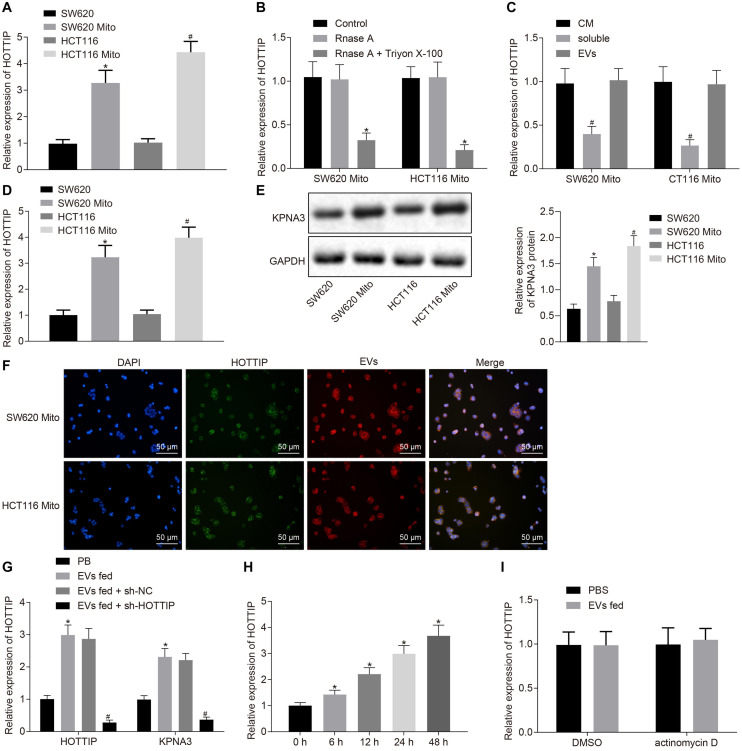
HOTTIP is transferred from the EVs derived from mitomycin-resistant cells to parental cells. **(A)** RT-qPCR analysis of the level of HOTTIP in the CM of parental and mitomycin-resistant cells. **(B)** CM of SW620Mito cells and HCT116Mito cells were treated with RNase A (2 mg/ml) alone or in combination with Triton X-100 (0.1%) for 20 min. The levels of HOTTIP in the CM were determined by RT-qPCR. **(C)** RT-qPCR analysis of the levels of HOTTIP in the derived EVs, CM supernatant and whole CM of mitomycin-resistant cells. **(D)** RT-qPCR analysis of the level of HOTTIP in CM of mitomycin-resistant and parental cells. **(E)** KPNA3 expression in mitomycin-resistant cells and their derived EVs measured by Western blot analysis. **(F)** Confocal microscopic observation of fluorescence signal (scale bar = 50 μm). **(G)** Determination of HOTTIP and KPNA3 expression in the parental cells co-cultured with EVs derived from the mitomycin-resistant cells by RT-qPCR analysis. **(H)** RT-qPCR analysis of HOTTIP expression in the parental cells co-cultured with EVs derived from the mitomycin-resistant cells at different time points. **(I)** The parental cells treated with actinomycin D (1 μg/mL) were co-cultured with EVs for 48 h. HOTTIP expression in the parental cells determined by RT-qPCR. *p < 0.05 vs. the SW620 cells, control, cells treated with PBS or cells co-cultured with EVs for 0 h; #*p* < 0.05 vs. the HCT116 cells, CM or parental cells co-cultured with EVs from the sh-NC-transfected mitomycin-resistant cells. Data (mean ± standard deviation) were analyzed by unpaired *t* test between two groups, and one-way ANOVA with Tukey’s *post hoc* test for multiple groups. The cell experiments were independently conducted in triplicates.

Exosomal HOTTIP might be a biomarker for cancer diagnosis and prognosis (Zhao et al., 2018). To detect the presence of HOTTIP in the extracellular environment, we extracted RNA from CRC cell culture medium (CM). Consistent with the up-regulation of HOTTIP in mitomycin-resistant cells (SW620Mito and HCT116Mito cells), the level of HOTTIP was higher in the CM of SW620Mito and HCT116Mito cells than in the CM of parental cells (SW620 cells and HCT116) (Figure 5A). Meanwhile, the HOTTIP level in CM remained unchanged after RNase treatment, but it was decreased when treated with RNase A and Triton X-100 simultaneously (Figure 5B), suggesting that extracellular HOTTIP was mainly coated by membrane rather than directly released.

Then, we purified EVs from CM, which was identified through electron microscopy, nanoparticle tracking analysis (NTA) and Western blot analysis. It was revealed that the separated extracellular vesicles (EVs) were indeed EVs and the EVs isolated from mitomycin-resistant cells and parental CRC cells had similar typical goblet morphology, size and number (Supplementary Figures 4A–C). Interestingly, there was no significant difference in HOTTIP levels between in EVs and in the entire CM (Figure 5C), indicating that EVs were the primary vesicles for extracellular HOTTIP. At the same time, as expected, HOTTIP levels were higher in mitomycin-resistant cells and their derived EVs than in parental cells (Figure 5D). In addition, Western blot analysis was performed to determine the KPNA3 expression in mitomycin-resistant cells and their derived EVs, and results revealed that KPNA3 was barely expressed in the EVs (Figure 5E).

After that, SW620Mito cells and HCT116Mito cells were transfected with fluorescein amide-labeled HOTTIP to observe the uptake of EVs. EVs were isolated from the CM of SW620Mito and HCT116Mito cells, which were then labeled by Dil. The parental cells were co-cultured with the purified EVs. Confocal microscopic images displayed the EV-encapsulated HOTTIP and internalization of EVs by the parental cells (Figure 5F). After co-culture of the parental cells with the purified EVs for 12 h, the expression of HOTTIP was up-regulated in the parental cells, and the expression of KPNA3 was also up-regulated. When sh-HOTTIP was introduced in parental cells, the expression of HOTTIP and KPNA3 was down-regulated (Figure 5G and Supplementary Figure 4D). Furthermore, the expression of HOTTIP was gradually increased in parental cells by prolonged co-culture with EVs (Figure 5H and Supplementary Figure 4E). Treatment with actinomycin D (RNA polymerase II inhibitor) did not alter the expression of HOTTIP in the recipient parental cells (Figure 5I and Supplementary Figure 4F) which excluded the possibility of endogenous induction of HOTTIP expression in the recipient parental cells. In conclusion, the above results indicated that HOTTIP was transferred from mitomycin-resistant cells to the parental cells via secreted EVs.

### Exosomal HOTTIP From Mitomycin-Resistant CRC Cells Induces Mitomycin Resistance

**FIGURE 6:**
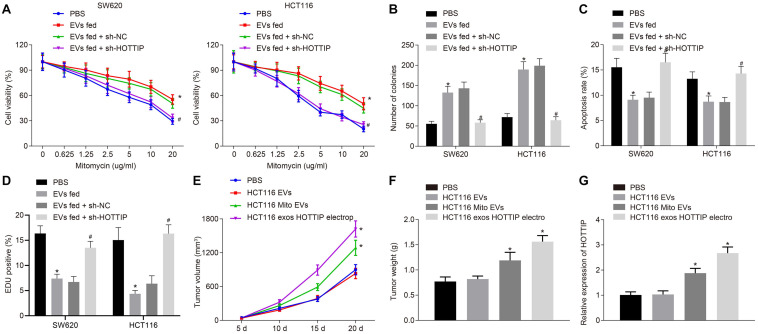
Exosomal HOTTIP confers the mitomycin resistance. **(A)** Viability of SW620 and HCT116 cells assessed by CCK-8 assay upon co-culture with HCT116Mito EVs and treatment with PBS, sh-HOTTIP and sh-NC. **(B)** Quantification of the number of colonies in SW620 and HCT116 cells upon co-culture with HCT116Mito EVs, and treatment with PBS, sh-HOTTIP and sh-NC. **(C)** Flow cytometric analysis of apoptosis rates of SW620 and HCT116 cells upon co-culture with HCT116Mito EVs and treatment with PBS, sh-HOTTIP and sh-NC. **(D)** Immunofluorescence analysis showing the γ-H2AX expression in SW620 and HCT116 cells. **(E)** Measurement of tumor volume. **(F)** Tumor weight in nude mice. **(G)** RT-qPCR analysis of the level of HOTTIP in tumor tissues of nude mice. *p < 0.05 vs. the tumor-bearing nude mice treated with PBS; #*p* < 0.05 vs. the tumor-bearing nude mice injected with EVs from sh-NC-transfected cells. Data (mean ± standard deviation) were analyzed by unpaired *t* test between two groups, and compared by one-way ANOVA with Tukey’s *post hoc* test among multiple groups. Data between groups at different time points was compared using repeated measures ANOVA with Bonferroni *post hoc* test. The cell experiments were independently conducted in triplicates. *n* = 5 in animal experiments.

We further investigated whether the HOTTIP transferred via EVs conferred a resistant phenotype of the parental cells. Parental cells were stably transfected with sh-HOTTIP after co-culture with the EVs isolated from mitomycin-resistant cells so that EVs were internalized in cells to exert their activity. After co-culture, the SW620 and HCT116 cells assessed by CCK-8 assay, flow cytometry, and colony formation assay. It was found that co-culture of EVs increased the IC_50_, enhanced the independent growth ability of anchorages, and reduced drug-induced apoptosis of the parental cells, but those effects induced by EVs were all reversed by knockdown of HOTTIP in the parental cells (Figures 6A–C). It indicated that HOTTIP transmitted mitomycin resistance through EVs. γ-H2AX immunofluorescence analysis showed that the parental cells co-cultured with EVs could attenuate the mitomycin-induced DNA damage to cells, which could be neutralized by stable knockdown of HOTTIP (Figure 6D). Finally, in order to determine whether HOTTIP affected the *in vivo* chemosensitivity, HCT116Mito and HCT116-derived EVs were injected into tumor-bearing nude mice. As depicted in Figures 6E–G, the EVs derived from HCT116Mito inhibited the response of xenografted CRC cells to mitomycin with increased HOTTIP expression in tumor tissues. In addition, the delivery of HOTTIP into the parental cell HCT116-derived EVs by electroporation also achieved a similar effect. Collectively, these findings indicated that EVs from mitomycin-resistant cells could confer the resistance to mitomycin by delivering HOTTIP.

### HOTTIP Is Predictive for Mitomycin Response in Patients With CRC

**FIGURE 7:**
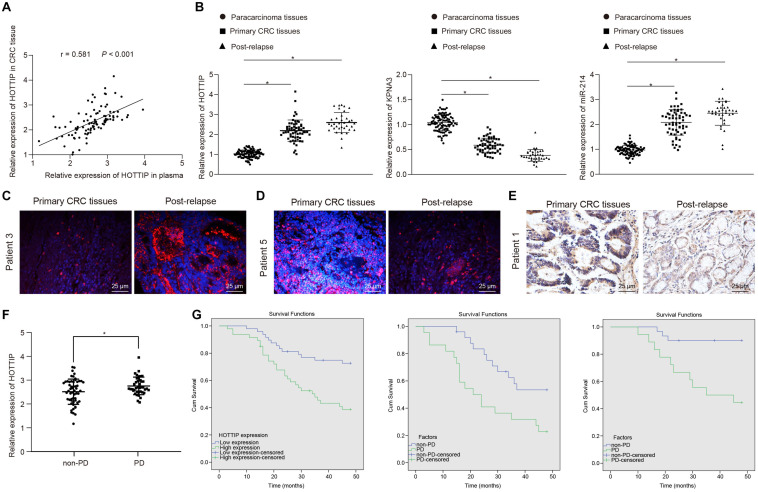
The levels of HOTTIP in plasma and tumor tissues are related to the response of patients with CRC to mitomycin. **(A)** Pearson correlation analysis of HOTTIP in plasma and cancer tissues of patients with CRC. **(B)** RT-qPCR determination of HOTTIP, KPNA3 and miR-214 levels in adjacent normal tissues, and cancer tissues from patients with primary CRC and those from patients with recurrent CRC who received mitomycin treatment. **(C)** FISH assay for the levels of HOTTIP in cancer tissues from patients with primary CRC and those from patients with recurrent CRC who received mitomycin treatment (scale bar = 25 μm). **(D)** FISH assay for the levels of miR-214 in cancer tissues from patients with primary CRC and those from patients with recurrent CRC who received mitomycin treatment (scale bar = 25 μm). **(E)** The level of KPNA3 in cancer tissues from patients with primary CRC and those from patients with recurrent CRC who received mitomycin treatment detected by immunohistochemistry (scale bar = 25 μm). **(F)** The levels of HOTTIP in the plasma of patients with PD and non-PD determined by RT-qPCR prior to mitomycin treatment. **(G)** Kaplan-Meier survival analysis of patients with high or low HOTTIP expression with the median HOTTIP level as the cutoff value. *p < 0.05 vs. adjacent normal tissues. Data (mean ± standard deviation) were analyzed adopting unpaired *t* test between two groups, and one-way ANOVA followed by Tukey’s *post hoc* test for multiple groups. *n* = 95 in clinical experiments. The cell experiments were independently conducted in triplicates.

To confirm whether HOTTIP was clinically relevant to human CRC development, we extracted RNA from the plasma and tissues of patients with CRC and found a positive correlation between HOTTIP expression in tumor tissues and that in the plasma of patients with CRC (Figure 7A). Kruskal–Wallis test analysis showed that the levels of HOTTIP and KPNA3 in the tumor tissues of patients with recurrent CRC who received mitomycin treatment were higher than those in tumor tissues of patients with primary CRC. Compared with adjacent normal tissues, the levels of HOTTIP and KPNA3 in CRC tissues were also increased. The level of miR-214 in tumor tissues of patients with recurrent CRC who received mitomycin treatment was lower than that in tumor tissues of patients with primary CRC, and miR-214 level in CRC tissues was decreased in comparison with adjacent normal tissues (Figure 7B). The results were further confirmed by fluorescence *in situ* hybridization (FISH) and immunohistochemistry (Figures 7C–E). The predictive value of lncRNA HOTTIP levels in the response of patients with CRC to mitomycin was subsequently examined. As shown in Figure 7F, the mean level of plasma HOTTIP was higher in patients with progressive disease (PD) before mitomycin treatment than in patients with non-PD before mitomycin treatment. Patients with CRC were grouped into high HOTTIP level group (*n* = 47) and low HOTTIP level group (*n* = 48) with a median HOTTIP level (2.311) as cutoff value. To determine whether HOTTIP expression in tumor tissues was associated with the clinical efficacy of mitomycin, we performed Kaplan-Meier survival analysis for patients in the two groups. As shown in Figure 7G, mitomycin treatment has limited benefit for the progression free survival (PFS) of patients with CRC. In a word, the above results suggested that HOTTIP could be used as an independent predictor of mitomycin response in patients with CRC.

## Discussion

Digestive malignancies are the dominating cause of death related to neoplasms which give rise to an tremendous burden on society in both economically developing and developed areas (Jiang et al., 2018; Shi et al., 2018). CRC is a heterogeneous disease and a clinical challenge (Wang et al., 2019). In spite of the fact that the treatment of CRC has yielded fruitful results, CRC remains a major cause of cancer-related death around the world and therefore potential therapeutic strategies are still necessary to be developed (Yamamoto and Mori, 2016). LncRNAs have been demonstrated to exert critical influence on cancer progression and development (Castro-Oropeza et al., 2018). In this current study, lncRNA HOTTIP was found to be highly expressed in mitomycin-resistant cells and could be transmitted from mitomycin-resistant cells to sensitive cells via EVs. HOTTIP up-regulated the expression of KPNA3 via impairing miR-214-targeted inhibition of KPNA3, and consequently the delivery of HOTTIP via EVs promoted mitomycin resistance.

First of all, the study suggested that HOTTIP was highly expressed in mitomycin-resistant CRC cells, and up-regulation of HOTTIP enhanced the mitomycin resistance. As demonstrated in a previous literature, HOTTIP, highly expressed in drug-resistant tissues, was able to promote the development of lung adenocarcinoma as well as to induce drug resistance (Zhang et al., 2017a). Interestingly, another study has also proved that knockdown of HOTTIP exerts an inhibitory function on prostate cancer cell proliferation and an accelerative effect on the sensitivity to cisplatin (Jiang et al., 2019). Besides, up-regulating HOTTIP accelerates osteosarcoma cell proliferation (Li et al., 2016). We also found that HOTTIP levels in plasma and tumor tissues were associated with the response of CRC to mitomycin and might function as an independent indicator predicting the efficacy of mitomycin. This was consistent with another finding that up-regulation of exosomal HOTTIP in the serum of patients with gastric cancer might be of good predictive value for prognosis (Zhao et al., 2018).

In addition, we provided evidence that HOTTIP could bind to miR-214 to elevate the expression of KPNA3. This lncRNA-miRNA-mRNA network is similar to another study which has suggested that HOTTIP up-regulates the expression of high mobility group B3 by binding to miR-615-3p in non-small cell lung cancer cells (Shi et al., 2019). Also, HOTTIP up-regulates the expression of B-cell lymphoma-2 through blocking the activity of miR-216a (Sun et al., 2018b). In addition, another research has suggested that EV-mediated transfer of lncRNA maternally expressed gene 3 decreases the level of miR-214 in ovarian cancer cells, thereby reducing drug resistance (Zhang et al., 2017b). Based on a former research, miR-214 enhances the sensitivity of breast cancer cells to Tamoxifen (Yu et al., 2015). Moreover, miR-214 has been proved to target Hsp27 to improve 5-fluorouracil sensitivity in colon cancer (Yang et al., 2019). More recently, KPNA3 has been identified to contribute to sorafenib resistance by inducing epithelial-mesenchymal transition in hepatocellular cancer (Hu et al., 2019). Additionally, lncRNA deleted in lymphocytic leukemia 1 promotes CRC progression by increasing KPNA3 (Liu et al., 2018b). In our study, HOTTIP was suggested to increase KPNA3 by inhibiting miR-214, which may underlie the molecular mechanisms conferred mitomycin resistance.

Furthermore, we observed in the present study that HOTTIP could be transferred from mitomycin-resistant cells to parental cells via exosomes, and exosomal HOTTIP disseminated mitomycin resistance. It is reported that lncRNA zinc finger antisense 1 could be delivered through exosomes to promote gastric cancer progression, which may serve as a potential diagnostic and prognostic biomarker for gastric cancer (Pan et al., 2017). In addition, exosomes-mediated transfer of lncRNA urothelial cancer associated 1 possess the ability to enhance tamoxifen resistance in estrogen receptor-positive breast cancer cells (Xu et al., 2016). Another report has demonstrated that lncRNA Activated in renal cell carcinoma with sunitinib resistance (lncARSR) can be delivered to sensitive cells via exosomes to disseminate sunitinib resistance, and it might act as a predictor and a novel therapeutic target for sunitinib resistance (Qu et al., 2016), which was similar to our findings that EV-mediated transfer of lncRNA HOTTIP conferred mitomycin resistance in CRC.

## Conclusion

**FIGURE 8:**
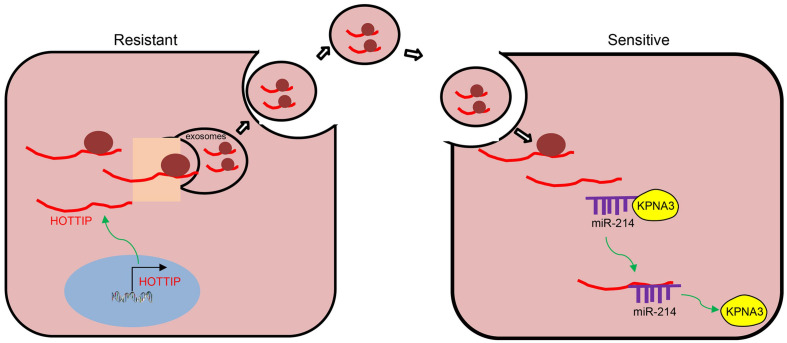
Schematic diagram of EV-carried HOTTIP functioned in the control of resistance of CRC cells to mitomycin. HOTTIP is highly expressed in mitomycin-resistant CRC cells and can be transmitted from mitomycin-resistant cells to sensitive cells via EVs. HOTTIP delivered into sensitive cells can compete with KPNA3 for binding to miR-214 to up-regulate the expression of KPNA3, and therefore promotes drug resistance in sensitive cells.

To sum up, we ascertained the promotive role of HOTTIP in mitomycin resistance in CRC. The expression of KPNA3 was potentially up-regulated by HOTTIP via binding to miR-214, and the EV-mediated transfer of HOTTIP induced drug resistance of CRC cells to mitomycin (Figure 8). Therefore, EV-delivered HOTTIP should be considered as a promising therapeutic target for the patients with CRC. Given that immune system plays a considerable role in the regulating resistance to therapies, studies should be performed to exclude the interference of immunomodulation on drug resistance in the future. Additionally, clinical experiments of fully developed therapeutic agent overcoming drug resistance should be conducted in the future.

## Data Availability Statement

The original contributions presented in the study are included in the article/Supplementary Material, further inquiries can be directed to the corresponding authors.

## Ethics Statement

The studies involving human participants were reviewed and approved by the Affiliated Tumor Hospital of Zhengzhou University. The patients/participants provided their written informed consent to participate in this study. The animal study was reviewed and approved by the Affiliated Tumor Hospital of Zhengzhou University.

## Author Contributions

XC, YL, and QZ designed the study. BL, YC, and YZ collated the data, carried out data analyses, and produced the initial draft of the manuscript. YS, JL, and HG contributed to drafting the manuscript. All authors have read and approved the final submitted manuscript.

## Conflict of Interest

The authors declare that the research was conducted in the absence of any commercial or financial relationships that could be construed as a potential conflict of interest.

## Footnotes

1 https://www.ncbi.nlm.nih.gov/

2 http://gepia.cancer-pku.cn/index.html

3 http://ophid.utoronto.ca/mirDIP/index.jsp#r

4 http://www.mirdb.org/

5 http://diana.imis.athena-innovation.gr/DianaTools/index.php?r=microT_CDS/index

6 http://www.microrna.org/microrna/home.do

7 http://www.rna-society.org/raid2/index.html

## Supplementary Material

Supplementary Figure 1 HOTTIP is expressed at high level in mitomycin-resistant CRC cells. **(A)** Data of capillary electrophoresis. **(B)** Migration and invasion of parental and mitomycin-resistant CRC cells assessed by Transwell assay. **(C)** Expression of γ-H2AX after treatment with mitomycin (30 μg/mL) for 24 h in parental and mitomycin-resistant cells by immunofluorescence staining. ^∗^*p* < 0.05 vs. the parental cells. Data (mean ± standard deviation) between two groups were analyzed by unpaired *t* test. The cell experiments were independently conducted in triplicates. E-cad, E-cadherin; N-cad, N-cadherin; Vim, Vimentin; Fn, Fibronectin.

Supplementary Figure 2 Overexpression of HOTTIP increases mitomycin resistance in parental cells. **(A)** HOTTIP expression in HCT116 and SW620 cells determined by RT-qPCR after HOTTIP overexpression. **(B)** Viability of HCT116 and SW620 cells after HOTTIP overexpression assessed by CCK-8 assay. **(C)** Colony formation rate in HCT116 and SW620 cells after HOTTIP overexpression. **(D)** Flow cytometric analysis of apoptosis rate of HCT116 and SW620 cells after HOTTIP overexpression. **(E)** Immunofluorescence analysis of γ-H2AX expression in HCT116 and SW620 cells. **(F)** Tumor volume in nude mice injected with HCT116Mito cells. **(G)** Tumor weight in nude mice injected with HCT116Mito cells. In **(F,G)** HCT116Mito cells (5 × 10^6^) transduced with lentiviral expressing Ctrl or HOTTIP were subcutaneously injected into the mice, and the mice were intravenously administered with mitomycin (8.8 mg/kg) once a week. ^∗^*p* < 0.05 vs. the cells transduced with lentiviral expressing Ctrl or the nude mice injected with cells transduced with lentiviral expressing Ctrl and NaCl; #*p* < 0.05 vs. the nude mice injected with cells transduced with lentiviral expressing Ctrl and mitomycin. Data (mean ± standard deviation) between two groups were analyzed by unpaired *t* test, and those multiple groups were compared using one-way ANOVA with Tukey’s *post hoc* test. Data between groups at different time points was compared using repeated measures ANOVA followed by Bonferroni *post hoc* test. The cell experiments were independently conducted in triplicates. *n* = 5 in animal experiments.

Supplementary Figure 3 KPNA3 knockdown attenuates the resistance of CRC cells to mitomycin. **(A)** KPNA3 expression in HCT116Mito cells infected with shKPNA3 determined by RT-qPCR. **(B)** Cell viability assessed by CCK-8 assay. **(C)** HCT116Mito cell colony formation rate. **(D)** Apoptosis rate of HCT116Mito cells detected by flow cytometric analysis. **(E)** KPNA3 overexpression efficiency in HCT116 cells determined by RT-qPCR. **(F)** Cell viability assessed by CCK-8 assay. **(G)** HCT116 cell colony formation rate. **(H)** Apoptosis rate of HCT116 cells detected by flow cytometric analysis. **(I)** miR-214 expression in HCT116Mito cells treated with miR-214 mimic determined by RT-qPCR. Data (mean ± standard deviation) between two groups were analyzed by unpaired *t* test. The cell experiments were independently conducted in triplicates. **p* < 0.05 vs. the cells in the shRNA group or the Ctrl group or the mimic-NC group.

Supplementary Figure 4 Exosomal HOTTIP derived from mitomycin-resistant cells is transferred to parental cells. **(A)** Representative electron microscopic images of EVs secreted from mitomycin-resistant and parental cells (scale bar = 100 nm). **(B)** Nanoparticle tracking analysis of the size distribution and number of EVs. **(C)** The expression of EV marker proteins CD63 and CD9 and *cis-*Golgi matrix protein GM130 in EV-enriched medium measured by Western blot analysis. **(D)** RT-qPCR analysis of HOTTIP and KPNA3 expression in the parental cells co-cultured with the exosomes isolated from mitomycin-resistant cells. **(E)** RT-qPCR analysis of HOTTIP expression in the parental cells co-cultured with exosomes at different time points. **(F)** The parental cells treated with actinomycin D (1 μg/mL) were co-cultured with EVs for 48 h. HOTTIP expression in the parental cells determined by RT-qPCR. **(G)** The transfection efficiency of miR-214 mimic in HCT116 cells and miR-214-sponge in HCT116Mito cells. **(H)** The transfection efficiency of KPNA3-pcDNA in HCT116Mito cells and siKPNA3 in HCT116 cells. ^∗^*p* < 0.05 vs. the cells with PBS or cells treated for 0 h. Data (mean ± standard deviation) were analyzed by unpaired *t* test between two groups, and one-way ANOVA with Tukey’s *post hoc* test for multiple groups. The cell experiments were independently conducted in triplicates. ^#^*p* < 0.05 vs. the cells in the exo fed group or the exo fed + sh-NC group.
