# Supplementary figures and images for: Exosomal Long Non-coding RNA HOTTIP Increases Resistance of Colorectal Cancer Cells to Mitomycin via Impairing MiR-214-Mediated Degradation of KPNA3
Source: Front Cell Dev Biol. 2021 Jan 28;8:582723. doi: 10.3389/fcell.2020.582723 (PMC7876302; doi:10.3389/fcell.2020.582723)

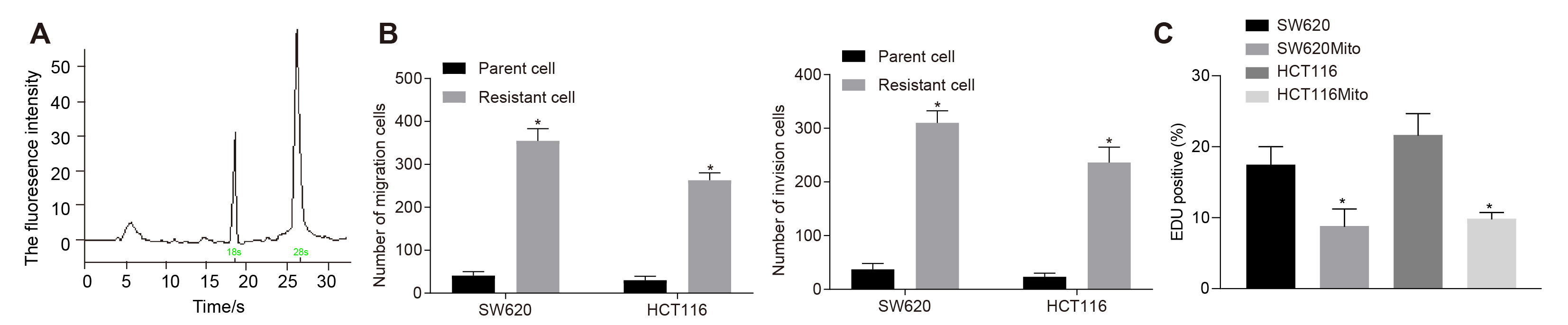

Supplement: Supplementary Figure 1 — HOTTIP is expressed at high level in mitomycin-resistant CRC cells. (A) Data of capillary electrophoresis. (B) Migration and invasion of parental and mitomycin-resistant CRC cells assessed by Transwell assay. (C) Expression of γ-H2AX after treatment with mitomycin (30 μg/mL) for 24 h in parental and mitomycin-resistant cells by immunofluorescence staining. ∗p < 0.05 vs. the parental cells. Data (mean ± standard deviation) between two groups were analyzed by unpaired t test. The cell experiments were independently conducted in triplicates. E-cad, E-cadherin; N-cad, N-cadherin; Vim, Vimentin; Fn, Fibronectin. [file Image_1.JPEG]

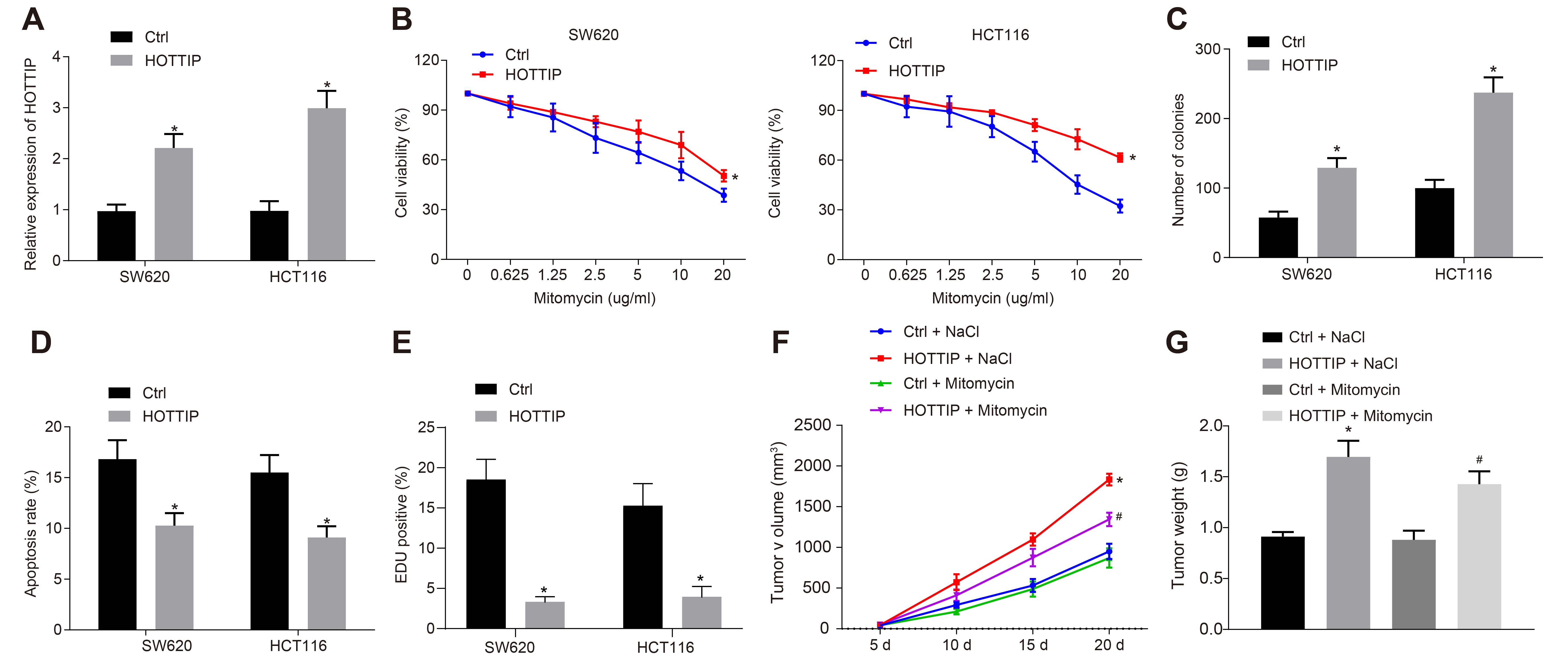

Supplement: Supplementary Figure 2 — Overexpression of HOTTIP increases mitomycin resistance in parental cells. (A) HOTTIP expression in HCT116 and SW620 cells determined by RT-qPCR after HOTTIP overexpression. (B) Viability of HCT116 and SW620 cells after HOTTIP overexpression assessed by CCK-8 assay. (C) Colony formation rate in HCT116 and SW620 cells after HOTTIP overexpression. (D) Flow cytometric analysis of apoptosis rate of HCT116 and SW620 cells after HOTTIP overexpression. (E) Immunofluorescence analysis of γ-H2AX expression in HCT116 and SW620 cells. (F) Tumor volume in nude mice injected with HCT116Mito cells. (G) Tumor weight in nude mice injected with HCT116Mito cells. In (F,G) HCT116Mito cells (5 × 10^6^) transduced with lentiviral expressing Ctrl or HOTTIP were subcutaneously injected into the mice, and the mice were intravenously administered with mitomycin (8.8 mg/kg) once a week. ∗p < 0.05 vs. the cells transduced with lentiviral expressing Ctrl or the nude mice injected with cells transduced with lentiviral expressing Ctrl and NaCl; #p < 0.05 vs. the nude mice injected with cells transduced with lentiviral expressing Ctrl and mitomycin. Data (mean ± standard deviation) between two groups were analyzed by unpaired t test, and those multiple groups were compared using one-way ANOVA with Tukey’s post hoc test. Data between groups at different time points was compared using repeated measures ANOVA followed by Bonferroni post hoc test. The cell experiments were independently conducted in triplicates. n = 5 in animal experiments. [file Image_2.JPEG]

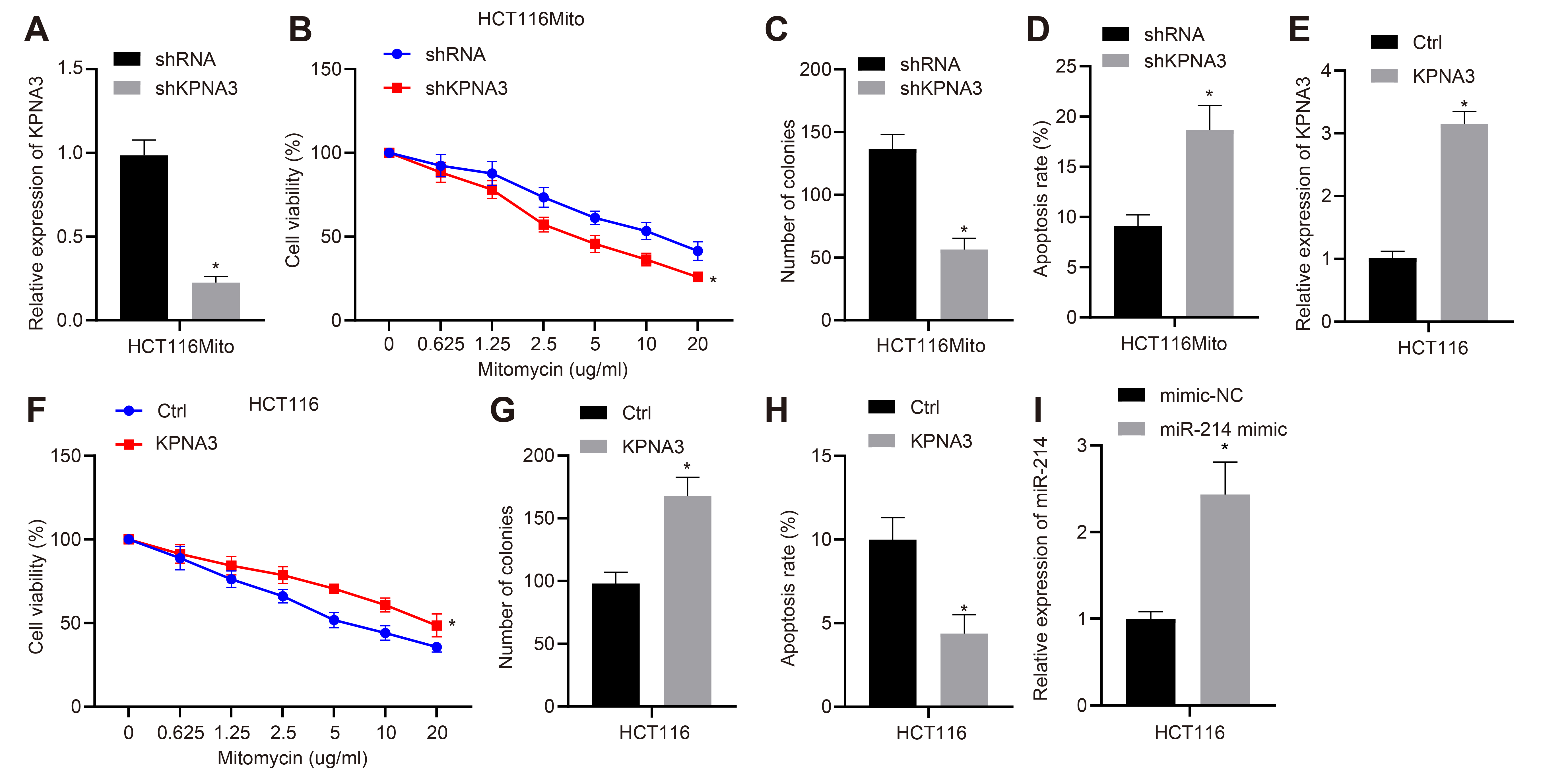

Supplement: Supplementary Figure 3 — KPNA3 knockdown attenuates the resistance of CRC cells to mitomycin. (A) KPNA3 expression in HCT116Mito cells infected with shKPNA3 determined by RT-qPCR. (B) Cell viability assessed by CCK-8 assay. (C) HCT116Mito cell colony formation rate. (D) Apoptosis rate of HCT116Mito cells detected by flow cytometric analysis. (E) KPNA3 overexpression efficiency in HCT116 cells determined by RT-qPCR. (F) Cell viability assessed by CCK-8 assay. (G) HCT116 cell colony formation rate. (H) Apoptosis rate of HCT116 cells detected by flow cytometric analysis. (I) miR-214 expression in HCT116Mito cells treated with miR-214 mimic determined by RT-qPCR. Data (mean ± standard deviation) between two groups were analyzed by unpaired t test. The cell experiments were independently conducted in triplicates. *p < 0.05 vs. the cells in the shRNA group or the Ctrl group or the mimic-NC group. [file Image_3.JPEG]

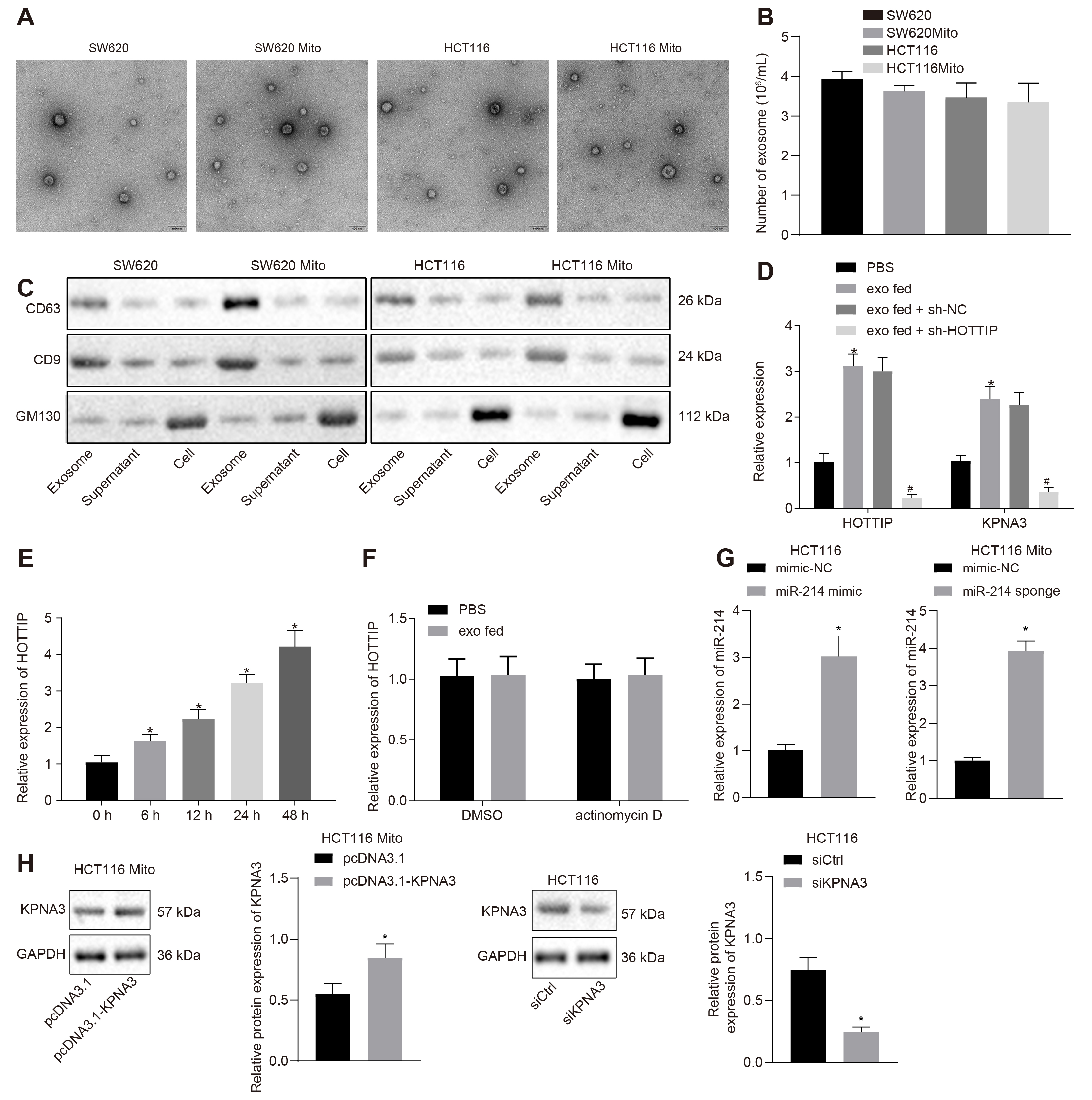

Supplement: Supplementary Figure 4 — Exosomal HOTTIP derived from mitomycin-resistant cells is transferred to parental cells. (A) Representative electron microscopic images of EVs secreted from mitomycin-resistant and parental cells (scale bar = 100 nm). (B) Nanoparticle tracking analysis of the size distribution and number of EVs. (C) The expression of EV marker proteins CD63 and CD9 and cis-Golgi matrix protein GM130 in EV-enriched medium measured by Western blot analysis. (D) RT-qPCR analysis of HOTTIP and KPNA3 expression in the parental cells co-cultured with the exosomes isolated from mitomycin-resistant cells. (E) RT-qPCR analysis of HOTTIP expression in the parental cells co-cultured with exosomes at different time points. (F) The parental cells treated with actinomycin D (1 μg/mL) were co-cultured with EVs for 48 h. HOTTIP expression in the parental cells determined by RT-qPCR. (G) The transfection efficiency of miR-214 mimic in HCT116 cells and miR-214-sponge in HCT116Mito cells. (H) The transfection efficiency of KPNA3-pcDNA in HCT116Mito cells and siKPNA3 in HCT116 cells. ∗p < 0.05 vs. the cells with PBS or cells treated for 0 h. Data (mean ± standard deviation) were analyzed by unpaired t test between two groups, and one-way ANOVA with Tukey’s post hoc test for multiple groups. The cell experiments were independently conducted in triplicates. #p < 0.05 vs. the cells in the exo fed group or the exo fed + sh-NC group. [file Image_4.JPEG]
